# Do Written Responses to Open-Ended Questions on Fourth-Grade Online Formative Assessments in Mathematics Help Predict Scores on End-of-Year Standardized Tests?

**DOI:** 10.3390/jintelligence10040082

**Published:** 2022-10-10

**Authors:** Felipe Urrutia, Roberto Araya

**Affiliations:** Centro de Investigación Avanzada en Educación, Instituto de Educación, Universidad de Chile, Santiago 8320000, Chile

**Keywords:** computational linguistics, online learning, student model, online formative assessments, student achievement

## Abstract

Predicting long-term student achievement is a critical task for teachers and for educational data mining. However, most of the models do not consider two typical situations in real-life classrooms. The first is that teachers develop their own questions for online formative assessment. Therefore, there are a huge number of possible questions, each of which is answered by only a few students. Second, online formative assessment often involves open-ended questions that students answer in writing. These types of questions in online formative assessment are highly valuable. However, analyzing the responses automatically can be a complex process. In this paper, we address these two challenges. We analyzed 621,575 answers to closed-ended questions and 16,618 answers to open-ended questions by 464 fourth-graders from 24 low socioeconomic status (SES) schools. Using regressors obtained from linguistic features of the answers and an automatic incoherent response classifier, we built a linear model that predicts the score on an end-of-year national standardized test. We found that despite answering 36.4 times fewer open-ended questions than closed questions, including features of the students’ open responses in our model improved our prediction of their end-of-year test scores. To the best of our knowledge, this is the first time that a predictor of end-of-year test scores has been improved by using automatically detected features of answers to open-ended questions on online formative assessments.

## 1. Introduction

One of the most recommended teaching and learning strategies is formative assessment (Black and Wiliam 2011; Hodgen et al. 2017). These are quizzes or low/no-stakes assessments. They are particularly recommended for the development of basic academic skills in elementary school students. Although the main audience of formative assessments is students, they are also critical for teachers (Gezer et al. 2021). They provide timely information to the teacher on the status of the learning process and an estimate of the state of knowledge attained by each student. However, it is important to distinguish between the knowledge attained immediately after learning activities and the definitive knowledge that will be revealed by students in the long term. There is a big difference between what the student demonstrates during or shortly after attending a lesson, and what she reveals months or years later. Estimating long-term achievement is a major challenge for the teacher. This is because there are many examples of strategies that generate learning when measured immediately after the intervention but demonstrate a rapid decline in the long run. There are also interventions where the opposite is true (Soderstrom and Bjork 2015). In these alternative interventions, students are exposed to a series of pre-designed and desirable difficulties (Bjork and Bjork 2020). These interventions require more effort from the student and lead to slower progress. Subsequently, students only manage a weak performance in the short term. However, they eventually produce a strong performance in the long term. Long-term learning in this kind of intervention is therefore better than in the first kind of intervention, which was described previously. This reversal phenomenon is difficult to swallow. According to Soderstrom and Bjork (2015), what we can observe and measure during teaching is performance, which is often an unreliable index of long-term learning. Soderstrom and Bjork (2015) make a critical distinction between performance, as measured during acquisition, and learning, as measured by long-term retention or transfer capacity. This is an unintuitive phenomenon, where fast progress generates an illusion of mastery in the students (Bjork and Bjork 2020). This illusion also hinders the teacher and makes it difficult for her to make a good prediction of each of her students’ long-term learning.

In this paper, we use a database of questions and answers taken from ConectaIdeas (Araya et al. 2015; Araya and Diaz 2020; Araya 2019; Araya and Cristia 2020). This is an online platform where students can answer closed- and open-ended questions. Teachers on ConectaIdeas can develop their own questions, designing them from scratch or taking them from existing material, or select them from a library of questions designed previously by other teachers. The teachers then use those questions to build their own formative assessments composed of sets of 20 to 30 questions. Students either answer them in laboratory sessions held once or twice a week or at home.

In principle, open-ended questions allow teachers to visualize their students’ reasoning. Additionally, promoting written answers to open-ended questions helps not only to reason about calculations but also to acquire written communication skills. This is particularly true if the question asks for explanations. These are questions that require more effort from students, but which are posed much less frequently than closed questions. Moreover, these are teacher-adapted or teacher-designed questions, thus leading to a wide range of question types. It is therefore more challenging to estimate their long-term learning than when working with questions taken from a carefully crafted and small list of closed questions. In this paper, we examine the responses of fourth-grade students from at-risk schools. The answers are very short. The average number of words in the responses is eight to nine words. This average increases as the school year progresses (Araya et al. 2018; Araya and Aljovin 2017; Araya and Diaz 2020). On the other hand, in previous RCT studies using the ConectaIdeas platform with low-SES fourth graders, we have found that the length of the responses to open-ended questions has a significant and positive effect on end-of-year learning in math (Araya and Diaz 2020).

Research question: To what extent do students’ short, written answers to teacher-designed, open-ended questions in weekly online formative tests help improve predictions of performance on end-of-the-year national multiple-choice standardized assessments?

## 2. Related Works

In this section, we examine related papers that investigate the possibility of making predictions of long-term learning and, on the other hand, the effect of answering open-ended mathematics questions.

Anozie and Junker (2006) developed linear predictors of student scores on end-of-year state tests using dynamic testing metrics developed from monthly log data taken from an intelligent tutoring system. They analyzed the data logs for 362 students, although only 105 students had complete data in each of the months. They found that logs from an online tutoring system provide better end-of-year predictions than using paper and pencil benchmark tests. They found that the adjusted $R^{2}$ is 0.637. However, all students attempted to solve a similar set of items. One of the challenges is that teachers prefer having the flexibility to adapt the exercises to their own experience. The authors therefore did not consider the situation in which teachers select or design their own set of exercises. Moreover, the study did not include answers to open-ended questions.

From a sample of 23,000 learners in Grades 6, 7, and 8 over three academic years, Zheng et al. (2019) analyze the relative contribution of different types of learner data to statistical models that predict scores on summative assessments. They use six different categories of statistical models to predict summative scores. One of the best model categories turned out to be Stepwise Linear Regression (SLR). In the best year, it achieved an $R^{2}$ of 0.734. However, this study does not consider variables associated with answers to open-ended questions. Apparently, the platform used does not include this type of question.

Dunlosky et al. (2013) analyzed 10 learning techniques and considered four categories of variables: learning conditions, student characteristics, materials, and criterion tasks. The techniques include elaborative interrogation, self-explanation, summarization, highlighting (or underlining), the keyword mnemonic, imagery use for text learning, rereading, practice testing, distributed practice, and interleaved practice. Two of these techniques are related to explaining. Elaborative interrogation is defined as generating an explanation for why an explicitly stated fact or concept is true. Self-explanation is defined as explaining how new information is related to known information or explaining steps taken. They found that self-explanation has a moderate effect and that a major strength is that its effects have been shown across a wide range of content materials and tasks. However, in their review, they do not study the effect of written explanations.

Rittle-Johnson et al. (2017) conducted a meta-analysis of the use of self-explanation to improve mathematics learning. These are self-explanations generated by the learner and without the intent of teaching to someone else. They reviewed 26 published studies that contrasted prompts to self-explain with a control condition involving unprompted explanations. They found a statistically significant effect (*p*-values less than 0.05) when testing is immediate but not after there is a delay.

Wang et al. (2021) propose a novel deep learning framework to effectively integrate both question explanations and student responses for predicting students’ current learning outcomes. They use the responses in two exams for training and try to predict responses in a third exam taken after the first two. Each exam has a total of 46 questions. The first two exams were taken by 5675 and 6895 students, respectively, while the third was taken by 26,330 students. In this sense, there was a small and fixed number of questions that were carefully designed by experts as well as a large number of students who answered them. This allows for patterns to be discovered using big data algorithms. In our paper, on the other hand, the questions are practically not repeated between different courses, since they are designed by the teacher. Although the teacher can copy them or adapt them from other teachers’ questions, they are very rarely copied verbatim. This condition is much more frequent and naturally occurring in classrooms, where the teacher decides on the spot to pose questions that they deem appropriate in that specific moment.

Bicer et al. (2013) studied the effect of writing on the mathematical problem-solving skills of 96 middle school students who participated in a 6-week afterschool program. The study compared the impact with a randomly assigned control group, who prepared for a high-stakes test involving mathematical problem solving. The authors found that students from the experimental group were more likely to generate and apply better problem-solving skills than the control group. Indeed, they outperformed the control group on a test of cognitive complexity and problem generation. It can therefore be inferred that there is some empirical evidence of the effect of writing on mathematics. However, for the control group, it is not clear whether they did practice tests using closed questions. In addition, the materials and the post-tests were designed by the research team. They were not end-of-year, state standardized summative tests.

McDaniel and Little (2019) compare the effects on learning of multiple-choice and short-answer quizzes. According to the authors, there is empirical evidence that more activated or engaged processes lead to improved retention on final summative tests. Multiple-choice tests rely more on recognition than recall, while laboratory studies have found that short answers lead to more long-term learning. However, their comparison study in a classroom setting found no clear advantages of short answers. This is a puzzling finding that contradicts the empirical evidence obtained in laboratory studies. In any case, short answers in this study only involved single-word responses. Students were not required to provide a sentence explaining a result. Instead, students needed to complete a sentence by suggesting the missing word.

Zhang et al. (2021) count the number of publications for student performance prediction per year from 2000 to 2020 from the perspective of machine learning and data mining. There is a sustained increase, and in 2019, there are six times the number of publications of the year 2000, reaching 60,000. The count considers offline as well as online classes. They examine various machine learning algorithms and test them on data from 1325 students and 832 courses. Data features include student grades, demographic features, social features, and school-related features. However, there are no features related to short answers to open-ended questions. They find that random forest is better for classification. The authors conclude that current studies are limited in statistical methods of feature learning.

López Zambrano et al. (2021) provide an overview of the current state of research in the end-of-year performance prediction. They study both online learning systems and traditional face-to-face learning. They identified 133 papers in the literature until November 2020. After several filters, they obtained 82 papers, most of them from 2018, 2019 and 2020. The most used methods for regression were logistic regression and linear regression. In online learning, the most used features are partial performance during the course. In the platforms, many features are used. Most of the used variables classified by educational environment and source of data are from activity on the platform. There are no features with answers to open-ended questions. The closest thing is the number of emails sent by students. The authors conclude that very little research has been conducted on early prediction in primary education. They consider that this is an open research area and that according to the results published in some recent papers, they estimate that the application of data mining techniques for early prediction of student performance in blended learning environments is a very promising area.

Pardos et al. (2014) include regressors related to affective states. In the type of written responses examined on the ConectaIdeas platform, incoherent written answers are a proxy for negative affective states. According to these authors, models that use a combination of student affect, disengaged behavior, and performance within the learning system achieve high overall correlation to standardized exam scores. They conclude that these types of features can effectively infer longer-term learning outcomes.

These papers show that the score at the end-of-the-year tests can be predicted with a reasonable degree of error and that written answers to open-ended questions help the learning process. However, in the prediction literature, there are not yet regressors related to the written answers to open questions (López Zambrano et al. 2021).

## 3. Materials and Methods

Our data come from a virtual platform called ConectaIdeas. This platform has a series of mathematics exercises for elementary school students. Each student answers two types of online questions during weekly sessions. The questions are created by teachers and can be either closed-ended (e.g., multiple choice questions) or open-ended (e.g., essay questions). Throughout the school year, students answer the 36 closed-ended questions and one or two open-ended questions during each session. The sessions are 90 min long, with two sessions held per week. Unlike the answers to closed-ended questions, the data for the answers to the open-ended questions are unstructured and provided in the form of a written text. This is a particularly challenging feature.

Below, we outline the materials and methods used to predicting the scores on an end-of-year national standardized test.

### 3.1. Predicting the Scores on the SIMCE End-of-Year National Standardized Test

#### 3.1.1. Data

We focused on using data gathered from the ConectaIdeas platform during 2017 from both open-ended and closed-ended online exercises. Fourth grade students used this platform to exercise mathematics content. Additionally, in order to measure progress in terms of performance when using the platform, 24 schools participated in an RCT study. In this study, only one of the two classes in each school worked on the platform (treatment group), while the other class did not have any access to the platform (control group). We will only focus on the responses given by students in the treatment group, since they are the only ones who answered both open- and closed-ended questions during the year.

Now, for simplicity, we only consider two important annual tests: one at the beginning of the year and the other at the end of the year. At the beginning of the year, a test was applied to measure the performance of students prior to participating in the study. This test is called the pre-test. The second test, called SIMCE, is a national standardized test that is conducted every year by an educational quality agency. We consider the pre-test per student as an estimator to predict the score on the end-of-year national standardized test (SIMCE). It is worth noting that at a national level, this standardized test has a mean of 260.95 and a standard deviation of 47.8, both of which are important reference values (Ulloa Miranda 2021).

Additionally, we include the average SIMCE score obtained by the school in 2016 as another estimator. Note that this estimator is at the school level and not per student. In addition, we automatically label responses to open-ended questions with two prediction models. The first is a question type detector, which consists of receiving a question and responding with one of six question types. These are: (1) Calculate without explaining, (2) Calculate with explaining, (3) Choice and/or affirmation, (4) Compare quantities, (5) Procedure and content knowledge and (0) Others. The second is a coherent answer detector, which receives both the answer and the question in order to detect whether or not the answer is incoherent. Answers that range from noisy text (e.g., laughter, faces and curse words) to nonsensical responses (e.g., answers a different type of question than required) are considered to be incoherent answers.

In the ConectaIdea platform, answers to open-ended questions do not receive a score according to the correctness of the answer to the open-ended question. However, for this study, years later, teachers labeled answers received in 2019 according to coherence, indicating whether the answer is incoherent or not. With this information, we fit a model capable of automatically predicting the coherence of written answers to open-ended questions. Then, we used that model to label the student answers received in 2017. In addition, coherence does not imply correctness, but the reverse is true. This information is used for two reasons: (1) Some of the incoherent answers (e.g., laughter, faces and curse words) are not appropriate for extracting linguistic features such as part-of-speech and dependency-tags. In addition, separating coherent answers from incoherent ones allows us to make sure that the answers are appropriate to the question asked and (2) incoherent answers also reveal a student’s animic, attitudinal, and not necessarily cognitive state. If the student responds with a scribble or something disjointed, it reveals a disrespectful, negative attitude or protest behavior. If he or she responds with something completely inappropriate, it is very different from making a calculation error or misexplaining a result by making conceptual errors. As Pardos et al. (2014) points out, affective variables are important for good prediction.

#### 3.1.2. Data Set Description

Below, we will perform a general analysis of the data for the prediction of 2017 SIMCE scores. In our case, we are interested in all those students on the platform who have answered at least one open-ended question (these are the written answers). In summary, only 58.29% of the students out of a universe of 796 students from across 24 schools have answered this type of question at least once. This corresponds to 464 students. Additionally, 44.59% of the 796 students are female. It can also be observed that neither the sex of the student nor the number of students with answers to open-ended questions are stable at the school level, since there are schools with more girls than boys and others with 78.04% of students with responses to open-ended questions.

Now, we filter by all students with at least one response to an open-ended question. When we reduce the sample to this universe of students, we obtain a more even distribution between male and female students (51.5% and 48.49%, respectively). It should be added that the 24 schools we studied correspond in general to at-risk schools, with a high percentage of vulnerability. For this, we use the index of vulnerability (IVE). This is an index that the Chilean Ministry of Education officially calculates for each school (database of IVE: https://www.junaeb.cl/ive, accessed on 16 March 2022). The weighted average of IVE (according to the number of students per school) for our data is 90.2%. The average of the IVE in Chile is 80.5% and the standard deviation is 17.2%. Thus, the IVE of these 24 schools is 0.56 standard deviations above the national mean. On the other hand, the average SIMCE score of the 24 schools in 2017 is 0.381 standard deviations below the SIMCE national average of that year. Despite this, there are students in some schools that stand out with 2.45 standard deviations above the national average.

Finally, to recall, in 2017, we have 1180 open-ended questions and 16,618 answers to this type of question. When compared with the number of answers to closed questions, we see that almost 36.4 times more closed questions are answered than open-ended questions. A student on average answers 35.81 open-ended questions during the year.

#### 3.1.3. Open-Ended Model

We collected all answers per student to both open-ended and closed-ended online questions. Answers to open-ended questions are text written by students in the ConectaIdeas digital platform. We will focus on building a model that uses the written responses to predict a final SIMCE score. The way students write their responses to mathematical problems has been studied and investigated as predictors of writing quality (Powell 1997). Other authors have studied the connection between writing style and students’ understanding of mathematical problems (Martin 2015). These studies have examined the previously unexplored territory between text and mathematical thinking (Pugalee 2001). A fundamental property of these responses is that they are digitally written texts, which is a feature that brings its own challenges, e.g., the detection of coherent answers given a question type. We use question type classifier and incoherent-coherent answer classifier to automatically tag our data. With this, we can filter the answers according to answer types.

On the other hand, several models have been tested for predicting a final score (Anozie and Junker 2006; Bicer et al. 2013; Namoun and Alshanqiti 2021). We consider a linear model for predicting scores, such as the models used by Ulloa Miranda (2021), Zheng et al. (2019) and Yamasari et al. (2021). For this, a student *i* is represented with a vector of *k* regressors $x_{i}=\left(x_{i,0},x_{i,1},\ldots ,x_{i,k}\right)$, and its estimated score is defined by:

$$\hat{\mathrm{score}}\left(b,w;i\right)=b+w_{0}\cdot x_{i,0}+w_{1}\cdot x_{i,1}+\ldots +w_{k}\cdot x_{i,k}$$

where the slope $w=(w_{0},w_{1},\ldots ,w_{k})$ and the intercept *b* are parameters of the fitted least squares regression with *n* students, that is:

$$\left(P\right)\;\left\{\begin{array}{cc} \mathrm{minimize} & \sum_{i=1}^{n}{\left({\mathrm{score}}_{i}-\hat{\mathrm{score}}\left(b,w;i\right)\right)}^{2}\\ s.t. & w\in R^{k},b\in R \end{array}\right.$$

where each ${\mathrm{score}}_{i}$ is the real score obtained by student *i*.

Now, traditional models use demographic and process variables as regressors for the student. This time, we are interested in using written responses to open-ended questions as regressors of the linear model. To do this, there are several challenges in working with unstructured data such as text. In particular, it is difficult to condense the information from various answers to different types of questions in different periods of the year into a one-dimensional representation. The simplest way is to consider only one response and retrieve attributes from it in order to construct variables in the linear model. Studies such as Nguyen et al. (2011) design a set of regressors from the text in order to predict a continuous variable. Others use teacher comments to predict student scores (Fateen and Mine 2021; Luo et al. 2015). Additionally, we deal with a series of responses and transform them into a useful predictor. For this, the following section is devoted to a regressor design based on written responses to open-ended questions. The model that uses this type of regressor will be referred to as an Open-ended model.

#### 3.1.4. Engineering and Selection of Regressors

We will now detail the methodology used to construct and select the regressors. The first stage consists of designing regressors that capture student information potentially useful for the prediction of their score. The second stage consists of selecting the most relevant variables for the prediction of the final score.

Indeed, for the construction of variables or regressors, we will separate the description into two distinct groups of variables. First, we will briefly describe those regressors that we called *Traditional*. Then, we will mention the challenges and solutions for the construction of regressors using the answers to open-ended questions, which we called *Based on written answers*.

*Traditional*. According to the study by Ulloa Miranda (2021), we will consider two groups of regressors:

- *Historical*. These correspond to variables that are expected to remain unchanged during the year. Among these are the grade, school, sex, SIMCE score of their school in the previous year, and school vulnerability index (IVE).
- *Dynamic*. Unlike the previous ones, these variables can vary during the year, e.g., number of online exercises answered on the first attempt. In this context, these variables are usually called Process variables (Zheng et al. 2019).

**Figure 1:**
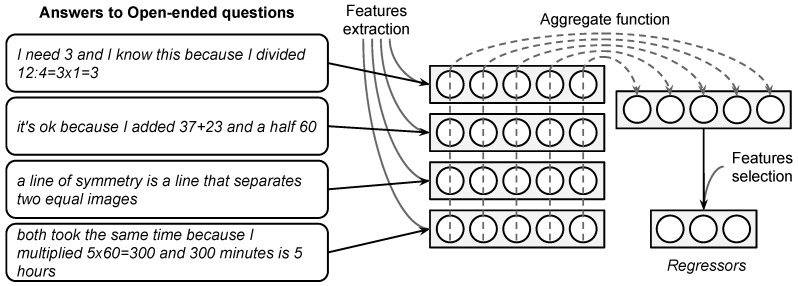
Description of the construction of regressors based on answers to open-ended questions (answers originally in Spanish).

*Based on written answers*. One of the big challenges is to capture relevant information in the written responses to build predictors of a final score. The simplest way to do this is to count how many such responses students make per year. However, this way of doing it omits valuable information in the words they use and the structure of students’ answers. On the other hand, not all answers are appropriate for this; e.g., in some cases, incoherent answers may be discarded. In addition, answers to certain types of questions have tokens of interest, e.g., numbers accompanied by units of measurement. We describe how we tackle this issue, following Figure 1.

Given a student, the main idea is to use all his/her answers (and respective questions). The objective is to transform all this information into a few relevant variables. For this, attributes are extracted from each of them independently (a state called Feature extraction). In this way, each student’s answer is encoded with a vector representation where each component of the vector is an interpretable variable. As each student answers more than one question, a stack of vectorized answers will be obtained (matrix, where each row is a vectorized answer). However, since each student answers a different number of questions per year, if we concatenate the rows of this matrix, we will obtain vectors of different lengths for each student. This is a problem when considering these as regressors for the linear model. To solve this, we will reduce the matrix to a single vector with the same number of columns, where each component condenses only the information of that column on individual vectors of the matrix (state called Aggregate function). Finally, as we wish to keep those components with the best predictions on the linear model, dimensionality reduction will be applied (a state called Feature selection).

In the following, we will detail the types of regressors based on written responses that we consider for the Open-ended model:

- *Simple indicators*. These are indicators that allow data to be aggregated: in our case, to aggregate the answers to open-ended questions. One indicator is the number, e.g., number of incoherent answers. Another one is the proportions, e.g., the proportion of coherent answers. In addition, there are double aggregate indicators, e.g., number of coherent answers to calculate with explaining questions.
- *Traditional/Semantic/Contextual features*. Different features can be extracted from any given answer. These are Traditional, Semantic and Contextual features. Traditional attributes are those that capture simple properties of the sequence of letters in words and the sequence of words in sentences, e.g., number of vowels and number of words. Semantic attributes, on the other hand, correspond to properties that have a conceptual charge in words, e.g., number of well-spelled words or curse words or emoticons. Meanwhile, Contextual attributes are those that depend on the environment of the sentence; in the case of answers, their context is the question, e.g., the number of similar words between the answer and its question. Given all the answers that are given by a student, the way in which these features are distributed is important, e.g., Average of number of words in coherent answers. These features can also be aggregated by question type, e.g., Standard deviation of number of numbers in answers to calculate without explaining questions.
- *Linguistic features*. Unlike the previous features, this time, we are interested in using linguistic knowledge to capture useful information from the answers. The most abstract features of an answer are (shape) the shape of the tokens and (alpha) indicator of whether the token is alphabetic. An example of this is the following: 
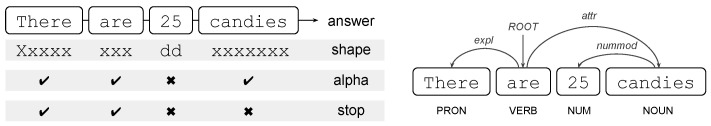
  Some words are noted for their abundance; these are called stop-words, e.g., {There,are} are of this type but {25,candies} are not. Shape, alpha and stop-word features are easy to detect, since in particular, they do not require information from the other tokens. However, they can condense valuable information about a student’s answers: for example, the feature Average of number of stop-words in answers to calculate with explaining questions.
  We will now consider two more attributes, but this time, they depend on the other words and need to be detected automatically. These are part-of-speech tagging (POS tags) and dependencies (dep), both of which are available in the Spanish version of the Spacy library (authors on https://explosion.ai/, accessed on 16 March 2022).
  POS tags are detected for each token in a response and can be: (PRON) Pronoun, (ADJ) Adjective, (VERB) Verb, and (NUM) Numeral, among others (Available in https://universaldependencies.org/u/pos/, accessed on 16 March 2022). An example of a regressor using POS tags is the following: Median number of verbs (tag/VERB) in coherent answers.
  Dependency tags correspond to syntactic dependencies between the tokens of the answer; e.g., if we remove candies from the response There are 25 candies then 25 will take the role of the root (dep/ROOT) of the sentence that had candies; Likewise, if we add candies then 25 obtains its characteristic of being a number with units, which is a dependency called a Numeric modifier (dep/nummod). An example of a regressor using dependency tags is the following: Average of number of dep/nummod in coherent answers. Other interesting dependency tags include: (obj) object, (nsubj) nominal subject, and (nmod) nominal modifier, among others (available in https://universaldependencies.org/u/dep/, accessed on 16 March 2022).

All of these feature-based regressors can be applied to a subset of responses and condensed into one dimension using specific functions. Further details of this are provided below:

- *Filters*. The simplest way to construct a regressor based on the answers is to consider all of them. Another way is to use a subset of the answers, e.g., only incoherent answers. This way of filtering is based on coherence. In addition, the type of question associated with the answer is automatically detected so that it can be grouped according to question type, e.g., only answers to questions of type Others. Likewise, both filters can be applied, i.e., both according to coherence and question type, e.g., only incoherent answers to questions of type Others. We will include several variables related to potentially incoherent written answers. Some attributes of these answers can reveal negative moods and attitudes of the student. However, negative affective states help improve the prediction of results in the end-of-year test (Pardos et al. 2014).
- *Aggregate functions*. We only focus on six functions on the features; these are: sum, average, standard deviation, minimum, maximum, and median. For example, if we select the attribute number-of-tokens in an answer, we can construct regressors by summing across all of the answers the number of tokens in the answer. If we want to see the expected value of the number of tokens, we just average the number of tokens. If the dispersion of this attribute is of interest, we use the standard deviation. For other attributes, it is useful to calculate the maximum, minimum, and median across all answers. Likewise, it is also possible to look at a subset of answers, e.g., minimum number-of-tokens in incoherent answers to calculate without explaining questions.

The following scheme is used for selecting regressors:

1. *Filtering*. All regressors with absolute correlation less than 0.19 are discarded. This is to avoid choosing regressors that are noisy.
2. *Genetic algorithm*. An algorithm that tests all combinations of regressors is impractical, since the number of candidates is exponential in the number of regressors. For this reason, and for simplicity’s sake, we rely on a genetic algorithm for selecting regressors. Based on the work of Leardi et al. (1992), the algorithm consists of the following stages: (0) Initial population; (1) Population evaluations; (2) Reproduction: select *k* individuals from the current population; (3) Mutation of the *k* individuals; (4) Cross-over between individuals and the current population; (5) Evaluation and selection of the fittest individual; and (6) Return to (1) and repeat the process. The algorithm starts with 14 regressors. These correspond to the regressors used by the Baseline model. For more details, go to Algorithm A1.
3. *Reduction*. Two things happen: (a) Too many regressors tend to be selected in relation to the number of regressors in the baseline model, and (b) linear models with more regressors tend to predict the observed variable better. To address this, we reduce the number of regressors selected until we obtain a similar number of regressors as the baseline model. For the Open-ended model, we choose the same number of regressors as in the baseline model to correctly compare the linear models with the $R^{2}$. For more details, go to Algorithm A2.
4. *Validation*. In order to avoid overfitting, we use 250 four-fold cross-validations.

#### 3.1.5. Evaluation

The following evaluation scheme is proposed to evaluate the model:

1. *Metrics*. For regression models, we use two statistical estimators to measure the model fitting. The first is the coefficient of determination or $R^{2}$, which is an indicator between 0 and 1 where closer to 1 is better. The second is the root mean square error or RMSE, which is an indicator that is on the scale of the observed variable where smaller is better. We will usually consider a normalized version of the RMSE in terms of the standard deviation of the observed variable at the national level (std SIMCE); simply divide RMSE with std SIMCE. The standard deviation corresponds to 47.80 according to the work of Ulloa Miranda (2021).
2. *Validation*. To validate, the *k*-fold cross-validation technique is used. This consists of randomly dividing the data set into *k* chunks of similar sizes. Training and testing with the entire data set allows having an estimate with *k* samples of the performance of a linear model more representative than an estimate with only one sample when using the classical approach. For the same reason, *N* random repetitions of *k*-fold cross-validation are performed; this corresponds to N×k fittings. Additionally, the data set is not randomly split so as to avoid two students from the same establishment being left in training and testing. To do so, a stratified randomization of the data is performed based on establishment, ensuring that no students to the same school are in different sets, thus avoiding data contamination (in our experiments, N=250, k=4).
3. *Baseline*. To compare the model based on open-ended questions, we propose a single baseline. As with the proposed model, the baseline is a linear regression model. This time, the regressors are the so-called traditional regressors, separated into historical and dynamic regressors. This model consists of 14 regressors: a pre-test, a double regressor corresponding to whether the student is male or female, a single socioeconomic regressor named school vulnerability index (IVE), 2016 SIMCE score for the establishment, and other regressors from the exercises completed during the year. In the work of Ulloa Miranda (2021), he proposes and studies each of the regressors of the baseline model. Of these, he designs 9 regressors called process variables: Number of exercises, Number of exercises answered at the first attempt, Number of exercises answered more than once, Differences between number of exercises answered at the first attempt and those answered more than once, Exercise accuracy (percentage of exercises solved on the first attempt), Grade point average for exercises, Number of answers to open-ended questions, Number of words in answers, and Average number of words per answer. Additionally, the baseline model mostly has variables in B-learning educational settings, such as age, gender, income, number of homework exercises, performance in weekly activities and final exam (López Zambrano et al. 2021).

## 4. Results

### Predicting the Score on the SIMCE End-of-Year National Standardized Test

Before showing the Open-ended model with its selected regressors, we will first show other regressors that use the answers to open-ended questions and that were not selected despite having a significant correlation between SIMCE score. Table 1 illustrates the importance of aggregating and filtering variables by question type and coherence, while Table 2 illustrates the advantage of variables using linguistic attributes. The correlation and *p*-value were obtained using the python scipy.stats module using the pearsonr function (documentation for pearsonr: https://docs.scipy.org/doc/scipy/reference/generated/scipy.stats.pearsonr.html, accessed on 16 March 2022).

First, Table 1 reports a series of regressors based on Simple indicators and Traditional/Semantic/Context features of the answer. Each of these are determined using different filters by answer type and different types of aggregate functions (see filters and functions in the regressor engineering section).

One of the most important regressors is probably the number of responses, with a correlation of 0.34 with the SIMCE score. However, if only coherent responses are considered, then the correlation improves to 0.43. A similar attribute is the proportion of coherent responses per student with a correlation of 0.42. On the other hand, if we only consider the answers to type 2 questions (calculate with explaining), then the average length of an answer has a correlation of 0.45 with the SIMCE score. This positive ratio indicates that students with a higher average answer length to type 2 questions obtain better scores. Another regressor with a strong correlation is the total amount of tokens that the student uses in their answers, with a correlation of 0.48. In this sense, the more tokens they use, the higher their SIMCE score. For other regressors, see Table 1.

We will now focus on the regressors that use the linguistic attributes of the responses to open-ended questions as an estimator of the SIMCE score. For simplicity, we will report only five of these in Table 2. However, there are several other regressors that use other POS (part-of-speech) and dependency tags from student sentences. For a careful comparison, we recommend looking at Table 1 at the same time. If we consider the number of numerical modifiers (dep/nummod) tokens in coherent answers to type 3 questions (choice and/or affirm), the correlation is 0.39. This is better than 0.30, which is achieved when only counting tokens in answers (see Table 1). The same is true if we count the number of numbers (tag/NUM) in the responses. Another important regressor is the average number of stop-words in the responses, which almost always improves the correlation instead of estimating the average number of words per response. For information on other linguistic attributes, please see Table 2.

Finally, the baseline model and the Open-ended model are subjected to the same evaluation criterion: 250 four-fold cross-validations stratified by establishment. For each model, the average value of the coefficient associated with the regressor and its relevance to the model at each training stage is determined. Both the correlation with the SIMCE score and these two values are reported in one table per model (Table 3 for the baseline model and Table 4 for the Open-ended model). In addition, after performing the 250 four-fold cross-validations, for each trained model, we can obtain the coefficients and plot their distribution. Visualizing the distributions of the coefficients allows us to observe the “robustness” of both models. In our case, we will observe two characteristics: the distribution of each coefficient should satisfy that (1) it is uni-modal with a small standard deviation and (2) the distribution does not cut to zero (change of sign of the coefficient). The results of linear models were performed through the statsmodels python library using the OLS function (documentation for OLS: https://www.statsmodels.org/dev/generated/statsmodels.regression.linear_model.OLS.html, accessed on 16 March 2022).

First, the baseline model has two regressors with the highest weight in the model. These are (1) Exercise accuracy (answers to closed questions), with a correlation of 0.76 with SIMCE and an average coefficient of 0.30 std SIMCE; (2) Average number of words per answer, with a correlation of 0.48 with SIMCE and an average coefficient of 0.29 std SIMCE. Historical variables such as sex and school vulnerability index are insignificant for the full model; i.e., they are not useful estimators when compared with process variables. To see other regressors, please see Table 3.

**Figure 2:**
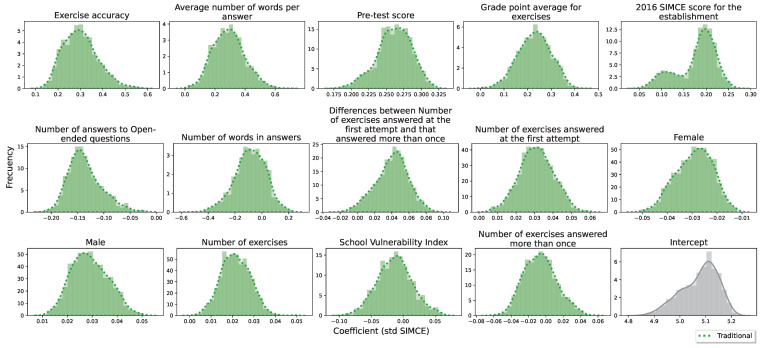
Distribution of each coefficient (std SIMCE) for the baseline model. These are obtained from 250 four-fold cross-validations. (Green) Traditional regressors.

According to Figure 2, the distribution of the coefficients is bell-shaped, except for the distribution of the coefficients of the SIMCE 2016 regressor that has two local maxima. Since it is the only school-level variable and the cross-validations are stratified at the school level, then its significance will depend on the few schools used for training. This can only explain why the distribution is not uni-modal. It is observed that four distributions cut zero, while only one of the four distributions cuts only slightly.

The Open-ended model has 14 regressors, just like the baseline model. Of these, only three correspond to regressors of baseline models. These are: Grade point average for exercises, Pre-test score, and 2016 SIMCE score for the establishment. These regressors are vital as basic regressors in a linear model, given their high predictive power. The difference is that the other 11 regressors of the Open-ended model report other student information that can be useful for making predictions. Of the 11 selected regressors, nine are the so-called linguistic attribute-based regressors, six filter for coherent responses and five filter for incoherent responses. They also filter answers by question type, where the question types chosen are types 1, 2 and 3. To see other regressors, please see Table 4.

**Figure 3:**
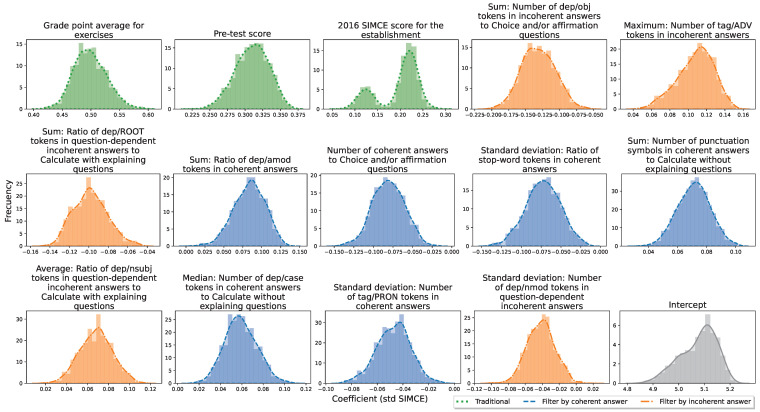
Distribution of each coefficient (std SIMCE) for the Open-ended model. These are obtained from 250 four-fold cross-validations. (Green) Traditional regressors. (Blue) Filter by coherent answers. (Orange) Filter by incoherent answers.

According to Figure 3, as with the baseline model, the only regressor with a non-uni-modal coefficient is SIMCE 2016. In addition, it can be observed that only one regressor cuts slightly to zero. The number of cuts is lower than those observed in the baseline model.

**Figure 4:**
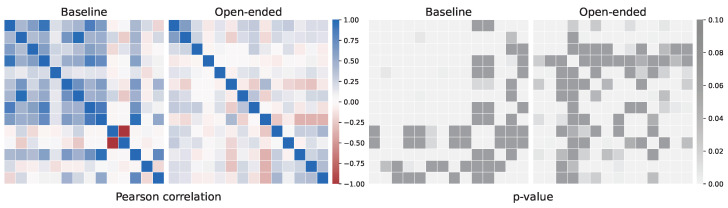
Correlation matrix between regressors. (**Left**) Pearson correlation with (**Right**) *p*-value.

Finally, we report the correlation matrix of both models (see Figure 4) and the evaluation metrics (see Table 5). The correlation matrix consists of a particular matrix in which the coefficient of row *i* and column *j* corresponds to the correlation between the *i*-th regressor and the *j*-th regressor.

First, the correlation matrix of the Open-ended model is more homogeneous and null than the that of the baseline model. This means that the Open-ended model regressors correlate less with each other than the baseline model regressors. This is a good feature if we want regressors with different informative estimators per student.

**Figure 5:**
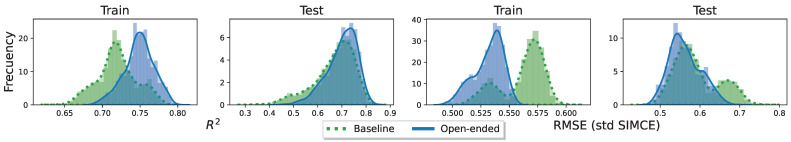
Distribution of $R^{2}$ and RMSE (std SIMCE) metrics for each model (in train and test sets). These are obtained from 250 four-fold cross-validations. (Green/dotted) Baseline model. (Blue/line) Open-ended model.

Next, the Open-ended model performs better in both training and testing than the baseline model, with an average $R^{2}$ of 0.70 versus 0.67 in the test set. Additionally, we consider a metric that indicates the proportion of times the Open-ended model is better than the baseline model in terms of $R^{2}$. In testing, 83.5% of the time, the Open-ended model is better than the baseline model in terms of $R^{2}$ (similar with RMSE). The value of the adjusted $R^{2}$ is also reported. See Table 5 and Figure 5 for details.

## 5. Discussion

### 5.1. Predicting the Score on the SIMCE End-of-Year National Standardized Test

We have built a set of predictors using written responses in addition to answers to closed questions. We use labels of predictions of question type and coherence type in order to filter the questions and obtain different regressors. Of all these regressors, the Open-ended model mostly uses only regressors based on linguistic attributes (9/14) and only three regressors of the baseline. The other two regressors of the Open-ended model correspond to a simple indicator and a regressor based on traditional attributes from the responses. In addition, all of the regressors selected for this model distinguish between response types according to coherence and/or question type. In particular, these regressors are better estimators for predicting a final score than regressors that do not distinguish between response types. In particular, five of these regressors only use responses that are detected as incoherent, with regressors for question types 1, 2 and 3 being predominant.

The baseline model and the model based on open-ended questions only share three of the regressors. The remaining regressors from the baseline model use simple indicators of written answers and estimators constructed using the closed-ended questions. This is not like the model based on open-ended questions, which uses the written answers to these types of questions and linguistic attributes from the answers.

Our results also show that the proposed model outperforms the baseline model. The $R^{2}$ of 0.67 from the baseline based on answers to closed questions rises to the $R^{2}$ of 0.70 from the Open-ended model. This is a statistically significant increase. It appears to be a modest increase. However, it is important to consider that the proportion of open-ended questions is very low. They are only 2.6% of all questions, while closed questions constitute 97.5% of the questions. This apparently modest increase in $R^{2}$ could increase substantially if the proportion of open-ended questions were much higher.

Some of the regressors of the Open-ended model do not contribute significantly to the prediction compared to the rest of the regressors; however, we observed with several experiments that removing them reduces the test performance.

We also found models based on open-ended questions with 30 regressors and an expected value of $R^{2}$ in test of nearly 0.75 and adjusted $R^{2}$ of 0.72. We even found models with 74 regressors and an expected value of $R^{2}$ in test of nearly 0.80 and adjusted $R^{2}$ of 0.74. We will analyze these models in a future study in which we will incorporate additional data from thousands of students who are currently using ConectaIdeas with open questions.

### 5.2. Regressors Related to Other Studies

Crossley et al. (2019) use linguistic features with NLP tools to automatically predict the quality of student summaries in English. The goal is to train a model that solves this task and can be used to inform online tutoring and feedback systems. Of the linguistic features, they retain 21 variables to then fit a generalized linear mixed model. Among the retained variables, they find two that measure the “syntactic complexity” of the students’ writing (Kyle 2016); these are “SD of dependents per nominal subject” and “SD of dependents per object of the preposition”.

The first one uses the nominal subject dependency tag (nsubj) and the standard deviation as the aggregation function. In our case, the variable “Average: Ratio of dep/nsubj tokens in question-dependent incoherent answers to calculate with explaining questions” considers this type of token but using the average as the aggregation function. In the referenced work Kyle (2016), they suggest that those students who use a wider range of nominal topic dependents in their essays tended to score higher.

The second variable uses the object dependency tag (obj) and the standard deviation as an aggregation function. In our case, the variable “Sum: Number of dep/obj tokens in incoherent answers to choice and/or affirmation questions” considers this type of token but using the sum as the aggregation function. In the referenced work Kyle (2016), they suggest that the best graded essays tend to include more dependents per object of the preposition.

On the other hand, among the retained variables, there is also one that measures the “lexical sophistication” of the students’ writing (Kyle et al. 2018), which is called “Frequency (COCA spoken, Function Words)”. This variable uses the frequency of “Function words” in the summaries, which are a type of words also known as stop-words. In the Open-ended model, the variable “Standard deviation: Ratio of stop-word tokens in coherent answers” considers this type of word but using the standard deviation as an aggregation function.

Crossley et al. (2018) examine the links between self-reported math identity, linguistic features extracted from student emails within an on-line math tutoring system, and student math score. To study the linkage, they perform a series of experiments with linear models. We will focus on only two of them; in the first experiment, they predict mathematical identity with linguistic features, while in the second experiment, they predict mathematical scores with linguistic features.

In the first experiment, they use a linear model with five linguistic features; two of them are named “Phonographic Neighbors: Function words” and “Word-naming accuracy: Function words”. These variables use the function words (stop-words) from the students’ mails to estimate the self-reported mathematical identity. In the Open-ended model, the variable “Standard deviation: Ratio of stop-word tokens in coherent answers” considers this type of word but using the standard deviation as the aggregation function. Crossley et al. (2018) in their results suggest that students who produce more sophisticated function words had a higher confidence in one’s mathematics ability related to one’s theory of self.

In the second experiment, they use a linear model with five linguistic variables; three of them are lexical indices, where one of them is called “Polysemy (adverbs)”. This variable uses the adverbs found in the emails sent and written by the students to predict their math scores. In the Open-ended model, the variable “Maximum: Number of tag/ADV tokens in incoherent answers” considers this type of word but using the maximum as an aggregation function. Crossley et al. (2018) suggest that the variable “Polysemy (adverbs)” indicates that students with higher math scores produce more sophisticated language, incorporating adverbs with fewer senses.

### 5.3. Interpretation of Regressors in the Open-Ended Model

In the following, we will give an interpretation of a subset of the variables in the Open-ended model. First, the first four most significant variables will be described in detail: that is, those variables with the third quartile ($q_{3}$) of the significant *p*-value. Second, we will mention three other variables that are not significant enough for our criteria but deserve a brief mention. We study in order the variables in Table 4.

#### 5.3.1. Sum: Number of dep/obj Tokens in Incoherent Answers to Choice and/or Affirmation Question

Incoherent answers to type 3 (Choice and/or affirm) questions can be either question-dependent or question-independent incoherent. As the proportion of independent incoherence to this type of questions is significantly less than the dependent ones, without loss of generality, the answers of interest fulfill two properties: (1) they are well structured answers, but (2) they do not answer the question asked. Thus, determining dependency between labels on responses that satisfy (1) and (2) is a potentially important feature.

Now, the dependency tag object (obj) of a verb is the second most important argument of a verb after the subject (definition of obj: https://universaldependencies.org/u/dep/obj.html, accessed on 16 March 2022). A token with this tag is the phrase that denotes the entity that undergoes the action given by the verb. An example of this type of tag is the following: 
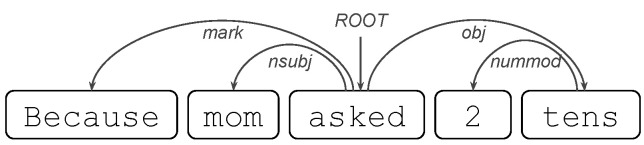
 where tens is the object that undergoes the movement of the verb asked.

On the other hand, if a student obtains a high value for this variable, it may be because (A) he/she answers several times with the obj dependency label or (B) he/she answers few times but in his/her sentences, there is repeatedly the presence of the obj dependency. Note that case (B) can be extended to case (A) by partitioning the long answers into short sub-answers. In any case, the answers have the characteristic of answering the request to “Explain”. They use a structure that attempts to justify a reasoning. However, they do not mention whether the reasoning is correct or not. This characteristic leaves them as incomplete answers that are therefore incoherent.

In summary, the variable quantifies incomplete answers to type 3 questions that have an appropriate structure. Therefore, higher values will punish more of those students who do not follow the requested question. Of course, this measure is with respect to the mean value and only for type 3 questions. Since the mean value is 0.58, it is reasonable to expect that students who have more than one token of this type will then decrease the value of their score even more.

#### 5.3.2. Maximum: Number of tag/ADV Tokens in Incoherent Answers

In general, incoherent answers, independent of the question, do not have an appropriate structure, since they are often silent words, sentences without logical meaning and/or non-words. For this reason, the presence of part-of-speech tokens should only be detected on incoherent and sophisticated answers, i.e., question-dependent incoherent answers. Now, the type of token of interest is the adverbial part of speech (ADV). These are words that usually modify verbs in the categories of time, place, direction or mood (definition of ADV: https://universaldependencies.org/u/pos/ADV, accessed on 16 March 2022). They can even modify adjectives and other adverbs. An example of this type of tag is the following: 
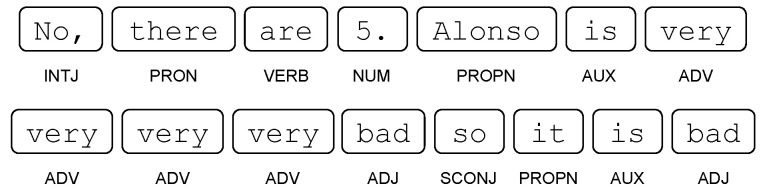
 where the word very repeatedly modifies the adjective bad.

As the variable is determined with a maximum, then the number of tokens of the ADV type corresponds to the count of adverbs on only one answer. Note that the correlation of this variable is positive (0.24 ***) and has a positive effect on the model. Therefore, if a student uses several tokens of the ADV type on one of his incoherent answers, he/she will have a directly proportional bonus in the estimation of his/her score. Since the presence of this type of label on sophisticated answers is often used as a discourse embellishment, the bonus is positive when trying to convince with his answer using a stylistic resource.

#### 5.3.3. Sum: Ratio of dep/ROOT Tokens in Question-Dependent Incoherent Answers to Calculate with Explaining Questions

This time, the responses of interest are incoherent-dependent responses to questions that ask both to calculate a quantity and to justify their answer. For the same reasons as before, the detection of dependency labels in this type of answers is appropriate. Now, the dependency label is ROOT. As the name implies, the ROOT grammatical relation points to the root of the sentence (definition of ROOT: https://universaldependencies.org/u/dep/root.html, accessed on 16 March 2022). Two examples of this type of tag are as follows: 
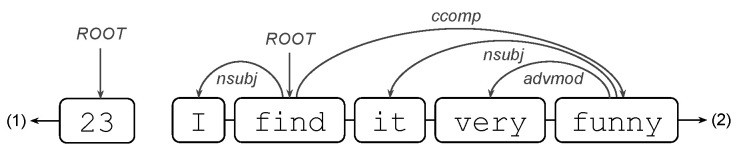
 where in (1), the only token 23 will have the ROOT tag, while in (2), the token will be find.

To understand the variable, we will first proceed by a description of ratio and then describe sum of ratios. First, the ratio of the ROOT tag in a response is the division between the number of tokens with the ROOT tag in the response and the number of tokens. Thus, since the root of a sentence is unique, then a high ratio is because the response is short. As expected, a ratio is low when the response is long. Second, the sum of the ratios of the ROOT tag among the responses is nothing more than the sum of the ratios of each response.

Thus, if this variable is high, it may be due to the fact that (A) he/she responds several times to short sentences or (B) few times, but in his/her sentences, there is repeatedly the presence of the ROOT dependency label. Note that case (B) can be extended to case (A) by partitioning the long answers into short sub-answers. In any case, the short answers are characterized by answering the request to “Calculate” but not “Explain”. They answer with only a quantity. However, they do not provide an explanation or justification of how they obtained their result. This characteristic leaves them as incomplete answers that are therefore incoherent. Unlike the longer responses, incomplete responses are penalized more than those that do not provide an amount.

Similar to the first variable described, this other variable quantifies incomplete answers to type 2 questions. Thus, higher values will penalize more those students who do not provide an explanation of how they obtained their result. Obviously, this measure is with respect to the mean value and only for type 2 questions. Since the mean value is 3.0, it is to be expected that students who have more than two short answers will further decrease the value of their score.

#### 5.3.4. Sum: Ratio of dep/amod Tokens in Coherent Answers

Unlike the previously described variables, this time, the variable to be analyzed only considers coherent answers to questions of all types. These types of answers are characterized by being well structured and responding to the underlying question. Note that the latter does not mean that the answer is correct. The tags used are adjectival modifier dependency (amod) tags. A token with this tag is any adjectival phrase that serves to modify the noun (or pronoun) (definition of amod: https://universaldependencies.org/u/dep/amod.html, accessed on 16 March 2022). An example of this type of token follows: 
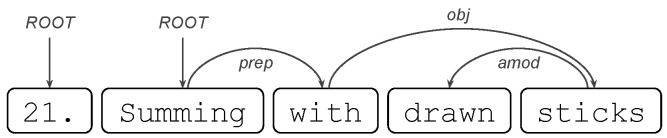
 where drawn is an adjectival modifier of the noun sticks.

As explained above for the sum of ROOT ratios, this time, a high ratio is due to the fact that the answer is short or has several amod tags. As expected, a low ratio is when the answer is long or has few amod tags. Subsequently, the sum of amod dependency label ratios among the responses is nothing more than the sum of the ratios of each response.

Note that the correlation of this variable is positive (0.28 ***), and with a positive effect on the model. Thus, if a student uses several tokens of the amod type on several of his coherent answers, he/she will have a directly proportional bonus in the estimation of his/her score. Since the presence of such labels in sophisticated responses is often used as a discourse ornament, the bonus is positive when adjectives are used as a stylistic device.

#### 5.3.5. Others

For the rest of the variables to be analyzed, the third quartile of the *p*-value of the coefficient is not significant. Under our criteria, they are not significant variables. However, it is worth describing them. Some of them have a clear interpretation. For others, their presence may be due to multiple causes that are outside the scope of the analysis in this paper.

*Number of coherent answers to choice and/or affirmation questions*. This variable is interesting, since it has a positive correlation with the variable to be predicted but contributes a negative effect on the model. This phenomenon is not uncommon. In fact, when working with linear models with more than one variable, there is no reason to expect the sign of the correlation to be the same as that of the model effect. The number of coherent answers has a positive effect but not so much in the type of choice and affirmation questions. Therefore, this regressor corrects for a possible overshoot of the number of coherent responses as accounted in the variable Sum: Ratio of dep/amod tokens in coherent answers.

*Standard deviation: Ratio of stop-word tokens in coherent answers*. Coherent answers are closer to being correct answers than answers that are incoherent. This is because, in particular, coherent responses answer the question appropriately, and incoherent answers do not. For this reason, determining linguistic features on these types of student responses captures structural properties that are not noise. Now, for the variable of interest, we determine the ratio of stop-words over all coherent responses. Stop-words are words that are abound in language. Some of these words are: the, if, what, not, of. Thus, (A) a high ratio of stop-words means that in the answer, there is an abundance of stop-words. Likewise, (B) a low ratio corresponds to a lack of stop-words. It is not clear which (A or B) is better. However, the contribution of this variable in the model suggests that if a student does not consistently follow either style A or B, then this weighs negatively on his/her score. Such consistency is measured by standard deviations. If the standard deviation is far from zero, then this explains an inconsistency of styles.

*Sum: Number of punctuation symbols in coherent answers to calculate without explaining question*. As mentioned for the previous variable, coherent answers are usually correct answers. This time, only coherent answers to questions that ask for a calculation but do not require a justification of how the result was obtained are important. Answers to such questions will be coherent if they at least contain an appropriate mathematical representation, e.g., 3/4. Such mathematical expressions are accompanied by punctuation symbols, e.g., /. In other cases, they serve to explain a procedure to obtain a result, e.g., 1/2 + 2 = 3/2. In this last case, four punctuation symbols are used, twice /, once + and once =. Other students usually represent the same mathematical objects but written in words, e.g., three quarters. This last example does not use punctuation symbols. However, since the variable is a sum of the total number of these punctuation symbols, then all the punctuation symbols used by the student are considered. Thus, students who represent their mathematical objects with symbols will also earn a bonus on their score. In addition, some students often explain using these symbols even though the question does not require them.

## 6. Conclusions

To the best of our knowledge, this is the first time a study has looked at the contribution of written responses to open-ended questions on weekly online formative assessments when predicting individual performance on end-of-year standardized mathematics tests by students in fourth grade.

Students only answered one or two open-ended online questions each week. This is 36.4 times less than the number of answers given to closed questions. Despite this, we found that the written answers provided information that allows us to make better predictions than when only working with responses to close-ended questions. In addition to there being very few open-ended questions when compared to closed questions, the students also wrote very short answers. These low-SES school students are not used to communicating in writing in mathematics. On average, the students wrote only eight to nine words for each answer. This is because they were not used to this type of question, let alone having to explain what they did. Even so, these written answers contain features that allow us to improve our predictions of long-term achievement, as measured by scores on a national summative test. The incredible thing is that this improvement in prediction is achieved even when the summative national tests do not contain any open-ended questions. This just shows the huge potential of open-ended questions and their written responses.

On the other hand, there is a growing interest in developing and implementing a dialogic pedagogy (Alexander 2006): that is, a didactic practice where the teacher promotes dialogue in class. Thus, the teacher provokes a constant reflection of each student and the exchange of arguments with him and among classmates. This didactic strategy is particularly weak in schools that serve the most vulnerable sectors of the population. It is in these sectors where there is more need to develop dialogic and argumentation skills. However, the challenge of getting everyone to exchange arguments at the same time is no small one. This is something that is not possible out loud, and doing it on paper is an inefficient alternative that generates more work for the teacher. Technology facilitates this simultaneous exchange. Everyone can respond in writing to the teacher. With online platforms, the management of answers to open questions and the subsequent peer review is very natural. The results obtained here in highly vulnerable schools support the fact that this is an option that can be implemented in practice. In addition, this strategy allows the teacher to improve the knowledge of the long-term learning achieved by each student. With the information from the answers to open-ended questions, each month, teachers have better predictions of what each of their students will achieve on the national end-of-year test.

In the near future, it would be important to look at the impact of an implementation with a larger number and proportion of open-ended questions. It would not be surprising if having students write more frequently and justify their responses were to have an even greater effect than that the one reported here. It also remains as future work to investigate how an early and automated warning of incoherence could speed up the process of producing coherent explanations as well as better explanations. On the other hand, it would be interesting to introduce instructions and explicitly teach students different strategies on how to explain their solutions to problems. We could then determine the effect this has on their adoption as well as on the students’ long-term learning. It also remains as future work to study the extent to which this process of written reasoning can be transformed from these early stages to a more rigorous form of mathematical reasoning, leading gradually to the sort of reasoning typically required in mathematical proofs.

## Figures and Tables

**Table 1:** Some interesting regressors using simple indicators and Traditional/Semantic/Context features of the answer. Correlation corresponds to the Pearson coefficient between the regressor and SIMCE score. The notation C0 corresponds to filter by coherent responses. The notation Q corresponds to filter by responses to Q-type questions.

Variable	All	C0	Q = 1	Q = 2	Q = 3	Q = 5	Q = 2 and C0	Q = 3 and C0
Average: Length of answers				.45 ***		.31 ***	.37 ***	
Average: Number of tokens in answers	.44 ***	.38 ***	.21 ***	.47 ***	.36 ***	.30 ***	.39 ***	.30 ***
Average: Number of tokens in answers and RAE dictionary				.48 ***		.29 ***	.40 ***	
Average: Number of tokens with at least one digit in answers	.44 ***	.39 ***	.08	.40 ***	.35 ***	.24 ***	.31 ***	.33 ***
Number of answers	.34 ***	.44 ***	.20 ***	.24 ***	.19 ***	.07	.33 ***	.28 ***
Proportion of coherent answers	.43 ***		.09 *	.34 ***	.29 ***	.15 **		
Standard deviation: Number of tokens in answers	.30 ***	.25 ***	.16 ***	.34 ***	.23 ***	.07	.33 ***	.23 ***
Sum: Number of tokens in answers	.48 ***	.49 ***	.31 ***	.42 ***	.36 ***	.20 ***	.42 ***	.38 ***

Note: * *p* < 0.05, ** *p* < 0.01, *** *p* < 0.001.

**Table 2:** Some interesting regressors using linguistic features of the answer. Correlation corresponds to the Pearson coefficient between the regressor and SIMCE score. The notation C0s correspond to filter by coherent responses. The notation Q corresponds to filter by responses to Q-type questions.

Variable	All	C0	Q = 1	Q = 2	Q = 3	Q = 5	Q = 2 and C0	Q = 3 and C0
Average: Number of stop-word tokens in answers	.47 ***	.42 ***	.26 ***	.47 ***	.39 ***	.30 ***	.40 ***	.33 ***
Standard deviation: Number of dep/nummod tokens in answers	.36 ***	.30 ***	.06	.28 ***	.34 ***	.04	.26 ***	.35 ***
Standard deviation: Number of tag/NUM tokens in answers	.37 ***	.32 ***	.07	.32 ***	.30 ***	.00	.29 ***	.30 ***
Sum: Number of dep/nummod tokens in answers	.47 ***	.49 ***	.18 ***	.34 ***	.37 ***	.19 ***	.34 ***	.39 ***
Sum: Number of tag/NUM tokens in answers	.50 ***	.52 ***	.21 ***	.39 ***	.37 ***	.19 ***	.40 ***	.40 ***

Note: * *p* < 0.05, ** *p* < 0.01, *** *p* < 0.001.

**Table 3:** Description of regressors for the baseline model. Correlation corresponds to the Pearson coefficient between the regressor and SIMCE score. Coefficient is the average value of the coefficient associated with the regressor divided by SIMCE standard deviation (47.80 reported in Ulloa Miranda 2021). Rank is the average value of the relative weight (less is better) of the modulus of the coefficient associated with the regressor for each fitted model. Both the Coefficient and the rank are obtained from 250 four-fold cross-validations.

Variable	Correlation	Distribution		Coefficient (Std SIMCE)		
		M±SD	Min/Max	M±SD	$p\;(q_{1}/q_{2}/q_{3})$	Rank
Exercise accuracy	.76 ***	0.59±0.11	0.23/0.85	0.30±0.08	** /*/ *	2±1
Average number of words per answer	.48 ***	136±79	4/541	0.29±0.10	*//	2±2
Pre-test score	.70 ***	39±19	4/96	0.26±0.02	***/***/***	3±1
Grade point average for exercises	.76 ***	5.5±0.6	3.7/6.7	0.23±0.07	** /*/	4±2
2016 SIMCE score for the establishment	.35 ***	230±19	207/274	0.18±0.04	***/***/***	5±1
Number of answers to Open-ended questions	.34 ***	36±11	5/60	−0.14±0.03	** /*/ *	6±1
Number of words in answers	.48 ***	300±167	8/1091	−0.09±0.12	//	7±3
Differences between Number of exercises answered at the first attempt and that answered more than once	.70 ***	261±376	−712/2602	0.04±0.02	//	9±2
Number of exercises answered at the first attempt	.59 ***	733±443	95/3152	0.03±0.01	//	10±1
Female	−.10 *	0.48±0.50	0.00/1.00	−0.03±0.01	*//	10±2
Male	.10 *	0.52±0.50	0.00/1.00	0.03±0.01	*//	10±2
Number of exercises	.44 ***	1205±583	157/3702	0.02±0.01	//	12±1
School Vulnerability Index	.02	0.90±0.06	0.75/0.97	−0.01±0.03	//	11±2
Number of exercises answered more than once	−.01	472±210	58/1565	−0.01±0.02	//	13±2
Intercept				5.08±0.07		

Note: *M* = Mean; *SD* = Standard Deviation. * *p* < .05, ** *p* < .01, *** *p* < .001; *q_i_* is the *i*-th quartile of *p*-value.

**Table 4:** Description of regressors for the Open-ended model. Correlation corresponds to the Pearson coefficient between the regressor and SIMCE score. Coefficient is the average value of the coefficient associated with the regressor divided by SIMCE standard deviation (47.80 reported in Ulloa Miranda 2021). Rank is the average value of the relative weight (less is better) of the modulus of the coefficient associated with the regressor for each fitted model. Both the Coefficient and the rank are obtained from 250 four-fold cross-validations.

Variable	Correlation	Distribution		Coefficient (Std SIMCE)		
		M±SD	Min/Max	M±SD	$p\;(q_{1}/q_{2}/q_{3})$	Rank
Grade point average for exercises	.76 ***	5.5±0.6	3.7/6.7	0.50±0.03	***/***/***	1±0
Pre-test score	.70 ***	39±19	4/96	0.31±0.02	***/***/***	2±0
2016 SIMCE score for the establishment	.35 ***	230±19	207/274	0.20±0.05	***/***/***	3±1
Sum: Number of dep/obj tokens in incoherent answers to Choice and/or affirmation questions	−.19 ***	0.58±1.03	0.00/5.00	−0.13±0.02	***/***/***	4±1
Maximum: Number of tag/ADV tokens in incoherent answers	.24 ***	1.4±0.9	0.0/60.0	0.11±0.02	***/***/**	6±1
Sum: Ratio of dep/ROOT tokens in question-dependent incoherent answers to calculate with explaining questions	−.25 ***	3.0±1.6	0.0/11.1	−0.10±0.02	**/**/*	7±2
Sum: Ratio of dep/amod tokens in coherent answers	.28 ***	0.38±0.29	0.00/2.02	0.08±0.02	** /*/ *	8±2
Number of coherent answers to Choice and/or affirmation questions	.28 ***	9.8±7.7	0.0/32.0	−0.08±0.02	*/*/	9±2
Standard deviation: Ratio of stop-word tokens in coherent answers	−.22 ***	0.22±0.05	0.00/0.43	−0.07±0.02	*/*/	9±3
Sum: Number of punctuation symbols in coherent answers to calculate without explaining questions	.30 ***	2.5±3.2	0.0/23.0	0.07±0.01	*/*/	9±2
Average: Ratio of dep/nsubj tokens in question-dependent incoherent answers to calculate with explaining questions	.29 ***	0.07±0.04	0.00/0.23	0.07±0.02	*/*/	10±2
Median: Number of dep/case tokens in coherent answers to calculate without explaining questions	.21 ***	0.43±0.52	0.00/2.00	0.06±0.01	*//	11±2
Standard deviation: Number of tag/PRON tokens in coherent answers	.21 ***	0.91±0.43	0.00/3.47	−0.05±0.01	//	13±1
Standard deviation: Number of dep/nmod tokens in question-dependent incoherent answers	.21 ***	0.45±0.25	0.00/1.42	−0.04±0.02	//	13±1
Intercept				5.08±0.07		

Note: *M* = Mean; *SD* = Standard Deviation. * *p* < .05, ** *p* < .01, *** *p* < .001; *q_i_* is the *i*-th quartile of *p*-value.

**Table 5:** Summary of $R^{2}$ and RMSE metrics for each model in training and test sets. These are obtained from 250 four-fold cross-validations. RMSE is divided by SIMCE standard deviation (47.80 reported in Ulloa Miranda 2021). The percentage symbol (%) corresponds to the proportion of times when the Open-ended model is better than the baseline model, with $R^{2}$ and RMSE resp. Support corresponds to the size of the set used for train or test, resp.

Set	$R_{\mathrm{adjusted}}^{2}$		$R^{2}$			RMSE (Std SIMCE)			Support
	Baseline	Open-Ended	Baseline	Open-Ended	%	Baseline	Open-Ended	%	
Test	.65±.09	.69±.07	.67±.08	.70±.06	83.5	.59±.06	.56±.04	83.5	116±13
Train	.71±.03	.74±.02	.72±.03	.75±.02	100	.56±.02	.53±.01	100	348±13

## Data Availability

Not applicable.

## Appendix A

### Appendix A.1. Genetic Algorithm to Feature Selection

This appendix describes in detail what the genetic algorithm used for the selection of variables (or regressors) of the Open-ended model consists of and how it is implemented. The Algorithm A1, is the main pseudocode of the genetic algorithm. The following sections define and explain each auxiliary function present in the pseudocode.

**Algorithm A1 (Main)** Genetic algorithm to feature selection	
**Require:**$x_{\mathrm{baseline}}:$ Vector of zeros and ones that indicate whether the regressor is in the baseline model, $n_{\mathrm{iter}}:$ Maximum number of iterations, D: Data (X,y) compatible with $x_{\mathrm{baseline}}$, I: Stratified indexes of *D*, with length p=|I|, of the form $\left[I_{1},\ldots ,I_{p}\right]$	
**Ensure:**(x,r)	
1: $\left(x,z\right)\leftarrow \left(x_{\mathrm{baseline}},\mathrm{SeedsGenerator}\left(I\right)\right)$	▹ Initial report station
2: m←Metrics({x},z,D,I)	
3: $r\leftarrow \mathrm{Average}\left(m_{x}\right)$	
4: (n,stop)←(0,False)	
5: **while** $n<n_{\mathrm{iter}}\wedge \neg \mathrm{stop}$ **do**	▹ Recursive stage
6: $s\leftarrow \mathrm{SelectIndividuals}\left(m_{x}\right)$	▹ Evolution station
7: mu←Mutation(x,s,z,D,I)	
8: co←CrossOver(x,mu)	
9: m←Metrics(co,z,D,I)	
10: $(x_{\mathrm{best}},r_{\mathrm{best}})\leftarrow \mathrm{SelectBest}\left(co,m\right)$	
11: **if** $r_{\mathrm{best}}\leq r$ **then**	▹ Update new status
12: stop←True	
13: **else**	▹ Report station
14: $\left(x,z\right)\leftarrow \left(x_{\mathrm{best}},\mathrm{SeedsGenerator}\left(I\right)\right)$	
15: m←Metrics({x},z,D,I)	
16: $r\leftarrow \mathrm{Average}\left(m_{x}\right)$	
17: n←n+1	
18: **end if**	
19: **end while**	

Broadly speaking, the algorithm is composed of two main stations: (R) reporting station and (E) evolution station. Each of these stations is used in key areas of the code. (R) is used to evaluate a model for a specific selection of variables, and (E) is used to modify a current selection of variables. To have a starting point, (R) is used. Then, a recursive process is performed that uses (E) to move forward and (R) to evaluate and decide whether to continue.

#### Appendix A.1.1. SeedsGenerator

The objective of variable selection is to find those variables that best generalize the linear model to a set of unobserved data. To quantify this notion of generalization, the data are traditionally divided into two disjoint groups of data, which are called training and testing; here, each set is used to train and test a model, resp. In our case, the way of dividing the data cannot be completely random, since some of the variables are not independent per student (e.g., the school’s SIMCE mean score in the previous year). A split that avoids this type of data contamination is called a stratified data split. The stratification we will use is by school; i.e., there cannot be students from the same school but in different training–test sets.

On the other hand, estimating the performance of a model is usually completed with a cross-validation using specific blocks of training and testing, which is a technique called k-fold cross-validation. This consists of dividing the data into *k* blocks, with similar sizes, where one of them is used as a test set and the rest is used for training. For a given partition, there are *k* ways to do this. To automatically construct each partition in a random way, we used the python library random with the function sample (documentation for sample: https://docs.python.org/3/library/random.html#random.sample, accesed on 16 March 2022).

In summary, the function SeedsGenerator receives a stratification of the students by establishment (*I*) and returns the N randomizations (or seeds) of the data, which are grouped by k blocks where one of them corresponds to testing. In our experiments, N=250, with k=4 emulating the typical division of 25/75 test/train. Thus, the output of the function is of the form $\left[z_{1},\ldots ,z_{N}\right]$, where each $z_{t}=\left[z_{t}^{1},\ldots ,z_{t}^{k}\right]$ has *k* splits of the data. Specifically, each $z_{t}^{q}$ is an array of zeros and ones of equal length to *I*, where those indices in $z_{t}^{q}$ with ones indicate whether such a stratification is used for testing, and it is zero otherwise (hence for training).

#### Appendix A.1.2. Metrics

To evaluate the performance of a model on a particular randomization of the data, a k-fold cross-validation is used to estimate this value. In the case of linear regression, the coefficient of determination, commonly denoted as $R^{2}$, is used. Since it is desired to evaluate performance in terms of generalization, this metric is used only on the test set.

Now, to evaluate the performance over any possible randomness of the data, it is necessary to compute the metrics over all potential randomness. In our case, we are reduced to estimate each quantity using the seeds generated by the function SeedsGenerator described in the previous part.

In summary, the function Metrics receives a set of regressor selectors, which is a collection of randomizations of the data where each of them corresponds to a possible k-fold cross-validation of the data. With this, the function returns the performance of the model (using a each regressor selector) on each collection and on each k-fold. For this, internally, the function Metrics queries a function that receives a k-fold cross-validation and returns the performance of a linear model trained and tested on each k-fold split. This function is called EvalModel, and it is defined in the next section.

#### Appendix A.1.3. EvalModel

In the previous section, we talked about estimating the performance of a model (defined by a regressor selector) on a particular randomness of the data using the k-fold cross-validation technique. Briefly, this consists of using the k splits of the data (proposed by the k-fold) and determining the $R^{2}$ of the linear model over each test set using its respective training set. Unlike a linear model fit with all variables, these will be filtered according to the regressor selector provided, i.e., training and testing the model with only a subset of the variables.

A regressor selector consists of a vector with zeros and ones of the same length as the total number of variables present in the data. Here, the components with ones represent which regressors are selected, and those with zeros represent those that are not selected. This is a vector *x* where {x=1} are the selected variables, and {x=0} are those that are not. For a data set D=(X,y), the variable selection simplifies to reduce *X* with those columns indented by {x=1}, that is, to eliminate those in {x=0}. We will denote $X^{x}$ as the selection of variables with selector *x*.

On the other hand, a k-fold cross-validation is of the form $z_{*}=\left[z_{*}^{1},\ldots ,z_{*}^{k}\right]$ with *k* splits of the data. Each $z_{*}^{q}$ is a split of the data in training and test. Those components in $z_{*}^{q}$ with ones indicate which subset of rows of $X^{x}$ is used for test and with zeros indicate those used for training, that is $\left\{z_{*}^{q}=1\right\}$ and $\left\{z_{*}^{q}=0\right\}$, resp. Similar with *y*. We will denote by $\left(X_{\mathrm{train}}^{x,q},y_{\mathrm{train}}^{q}\right)$ and $\left(X_{\mathrm{test}}^{x,q},y_{\mathrm{test}}^{q}\right)$ those portions of the data filtered according to $z_{*}^{q}$.

According to a typical preprocessing, the data (both training and test) are transformed using standardization, i.e., translated with the mean and scale with the standard deviation, both with the training data. For this, we use from the python module sklearn.preprocessing the function StandardScaler (documentation for StandardScaler: https://scikit-learn.org/stable/modules/generated/sklearn.preprocessing.StandardScaler.html, accesed on 16 March 2022). The preprocessed training and test data, respectively, will be denoted by ${\hat{X}}_{\mathrm{train}}^{x,q}$ y ${\hat{X}}_{\mathrm{test}}^{x,q}$. For interpretability, the variable *y* is not standardized in the process.

After pre-processing the data, a linear model is trained with $\left({\hat{X}}_{\mathrm{train}}^{x,q},y_{\mathrm{train}}^{q}\right)$. Subsequently, this model is evaluated according to $R^{2}$ with $\left(y_{\mathrm{pred}}^{q},y_{\mathrm{test}}^{q}\right)$. Here, $y_{\mathrm{pred}}^{q}$ corresponds to the prediction of the model with ${\hat{X}}_{\mathrm{test}}^{x,q}$. To achieve this, we used the python module sklearn.linear_model with the function LinearRegression (documentation for LinearRegression: https://scikit-learn.org/stable/modules/generated/sklearn.linear_model.LinearRegression.html, accesed on 16 March 2022).

In summary, the function EvalModel receives a regressor selector *x*, a stratification of the data (*I*), the data D=(X,y) and a possible k-fold cross-validation of the data. With this, the function returns the value of $R^{2}$ on test over each k-fold split. This value is determined using the model that chooses those variables selected by *x*, which is trained with the preprocessed training blocks and tested in the test block. This is the same procedure as described in the previous paragraphs.

#### Appendix A.1.4. SelectIndividuals

For a regressor selector (*x*) and a seed collection of data (*z*), the metrics obtained over each k-fold $z_{t}$ in *z* can be averaged. We denote this value by ${\bar{m}}_{x}^{t}$. Given the randomness of seed choice, the values of each ${\bar{m}}_{x}^{t}$ are almost certainly different. Potentially, there exist k-folds $z_{t}$ such that ${\bar{m}}_{x}^{t}$ is maximum. However, these values are optimistic, since they do not fully represent the performance of the model defined by *x* over any randomness in the data. At the same time, there exist k-folds $z_{t}$ such that ${\bar{m}}_{x}^{t}$ is minimum. By the same argument, this time, these values are pessimistic, since they are not representative. Thus, the random choice of some $z_{t}$ will tend to be a better representative of the actual distribution of expected value of performance $E({\bar{m}}_{x}^{t})$. However, the choice of one $z_{t}$ of each type (optimistic, pessimistic and random) summarizes globally the performance of the model in any of the possible cases, i.e., best, worst, and normal case, respectively.

Now, as each ${\bar{m}}_{x}^{t}$ was defined, its value is conditionally dependent on the model that uses the regressors selected by *x*. That is, if *x* is modified (e.g., adding regressors), there is a non-zero probability that $m_{x}^{t}$ will change value, and potentially, this value will increase. Following this idea, each possible case for ${\bar{m}}_{x}^{t}$ (best, worst and normal) characterizes a starting point for modifying the variables selected by *x*. Under certain conditions, the random choice of only the normal case could increase the number of iterations to find an optimal set of variables. This hypothesis is no more than a design assumption; this paper does not study whether this hypothesis is true or false.

Another view of the choice of only three cases, defined as best, worst, and normal case, follows from informative assumptions Leardi et al. (1992). A worst case is potentially more informative than a normal case. This is because the associated seed describes a case where the variables selected by *x* do not perform well. On the other hand, for the best case, the information loaded by the variables selected by *x* are valuable, since they allow a good performance. As this performance of the selected variables may not be representative, the normal case regularizes the performance to the possible superfluous performance of the worst and best case.

In summary, the function SelectIndividuals receives the metrics obtained by a linear model (with a subset of selected variables) on a randomization of the data. With this, three indices are returned corresponding to the seeds (or k-fold) where the model obtained the best-, worst- and normal value of the average $R^{2}$ over the k-fold.

#### Appendix A.1.5. Mutation

As suggested in the previous section, given a regressor selector (*x*) and a possible k-fold cross-validation of the data, *x* can be modified such that the metrics improve over the k-fold. It is not clear how to perform a clever modification of the regressors selected by *x*. This is due to the number of possible variants of a selection of variables, since this is exponential in the total number of variables. There are several ways to modify *x*, among these are: adding an unselected variable, removing a selected variable, replacing a selected variable with unselected variables, versions of the above operations but with pairs of variables, etc. In this work, we reduced ourselves to use only two operations, adding and removing variables.

The procedure chosen using these operations to modify a regressor selector to a better one consists of a recursive process that interleaves operations that add and remove variables in such a way that a variable is added as long as the previous performance is improved, analogously with the operations of removing variables. This procedure eventually ends, since the number of variables is finite, so at most, the attribute selector adds all variables or is left with at least one variable. These are cases where the model achieves over-fitting or under-fitting, respectively.

Now, testing all combinations of finding a variable in which adding (or removing) it will improve performance consumes a considerable amount of time. Therefore, at each iteration, the algorithm randomly chooses a subset of candidate variables to add (or remove, respectively). In our experiments, we consider a subset of size at most 20 variables. This simplification is equivalent to the probability of mutation defined by Leardi et al. (1992). To automatically construct each subset in a random way with a specific size, we used the python library random with the function sample. To evaluate the performance of the model when adding (or removing) a variable, the already defined function EvalModel is used.

In summary, the function Mutation receives a selector of regressors (x) and a set of k-folds (or individuals) where the modifications will be performed. It returns the mutations of *x* using each individual according to the procedure described in the previous paragraphs, i.e., a recursive process of adding–removing variables with random subsamples.

#### Appendix A.1.6. CrossOver

So far, we have one regressor selector (*x*), or parent, and three regressor selectors ($x_{\mathrm{best}},x_{\mathrm{worst}}$ and $x_{\mathrm{normal}}$), or potential offsprings. Here, each potential child corresponds to the modified version of *x* with mutations on its associated k-folds (best, worst, and normal case) obtained with the function Mutation defined in the previous section. If we select any of the potential offsprings as the next generation, the selected regressors are likely to encode information biased to the associated seed that was used for the mutation, which only saves local information from the data. This is because at that stage, the variables selected are such that the performance is maximal. To avoid bias in the decision of the candidate offsprings, a cross-over is performed.

When the mutation step is performed, two things usually happen: (a) between each potential offspring, the variables chosen are mostly different and (b) the same, but between each potential offspring with the parent. This is a problem, since the information from each potential offspring and parent is equally valuable. However, it is necessary to choose a single offspring that retains the most information from both the potential offsprings and the parent. To solve this, the potential offsprings can be mixed with each other and with the parent. This technique serves both to regularize the optimization and to explore other untested regressor selectors.

Now, to explain how to mix regressor selectors, we will consider $x_{1}$ and $x_{2}$ as two example regressor selectors. Suppose $x_{1}$ and $x_{2}$ are different but share at least one selected variable. If $x_{1}$ and $x_{2}$ share a selected variable, then the mixture also selects it, i.e., the common information holds. If $x_{1}$ selects a variable that $x_{2}$ does not select, then with probability *p*, the mixture also selects that variable. This allows the mixture to be randomized without privileging the origin of the variable and avoid that indiscriminately adding all the variables. If $x_{1}$ matters as much as $x_{2}$, then the probability *p* is equal to 0.5. However, in our experiments, we consider p=0.7, privileging $x_{1}$. This process repeats itself, but with probability 1−p when $x_{2}$ selects a variable that $x_{1}$ does not select.

With this scheme, we can mix two regressor selectors. For simplicity, we will consider this way of mixing and not with trios, quartets, etc. On the other hand, for four regressor selectors (a parent and three potential offsprings), there are six possible pairs of regressor selectors. These are $P_{1}=\left(x,x_{\mathrm{best}}\right),P_{2}=\left(x,x_{\mathrm{worst}}\right),P_{3}=\left(x,x_{\mathrm{normal}}\right),P_{4}=\left(x_{\mathrm{best}},x_{\mathrm{normal}}\right)$, $P_{5}=\left(x_{\mathrm{best}},x_{\mathrm{worst}}\right)$ and $P_{6}=\left(x_{\mathrm{normal}},x_{\mathrm{worst}}\right)$. Then, each pair of regressors can be mixed, obtaining the following six new regressor selectors $co_{i}=co\left(P_{i}\right)$. Here, for each i=1,…,6, $co(P_{i})$ corresponds to the cross-over (or mixing) between the regressor selectors in $P_{i}$.

In summary, the function CrossOver receives a set of three regressor selectors (or potential offspring) and a reference regressor selector (or parent), which will be mixed together. The function returns six new selector regressors, which correspond to the cross-overs between each potential offspring and between each potential offspring with the parent. The mixing (or cross-over) procedure follows the logic described in the previous paragraphs, i.e., keeping common information and randomly withholding information that is not common.

#### Appendix A.1.7. SelectBest

After performing a cross-over, one of the new selector regressors (or new individuals) must be chosen to become the offspring (or new parent). To do this, given the seed collection of data (z), the performance of each new individual on *z* is evaluated. The performance is calculated with the already defined function Metrics. Then, for each new individual, we average all its local performance ($R^{2}$ in each k-fold), estimating the global performance over *z*. With these values, the individual with the highest global performance is chosen.

In summary, the function SelectBest receives a set of metrics associated to cross-over individuals over a seed collection of data and returns the individual with the best global performance.

### Appendix A.2. Reduction Algorithm to Feature Selection

This appendix describes in detail what the Reduction algorithm used for the selection of variables (or regressors) of the Open-ended model consists of and how it is implemented. The Algorithm A2 is the main pseudocode of the Reduction algorithm. The previous sections define and explain each auxiliary function present in the pseudocode.

The purpose of this algorithm is to reduce the number of regressors selected to an equal number of regressors as those used for the baseline model. For this, the algorithm consists of two stages: (1) initialization stage and (2) recursive stage.

The initialization stage corresponds to start with a regressor selector ($x_{0}$) and determine the number of variables already selected $n=|\{x_{0}=1\}|$. Then, in the recursive stage, each selected variable is tested to determine which to remove. This decision is made by globally generating a seed collection of the data (*z*) and estimating the performance of a linear model without one of the selected variables. To compute *z*, we use the already defined function SeedsGenerator. Then, the selected variable that reduces the performance the least is removed from the current regressor selector. The procedure is repeated until a number of $n_{\min}$ regressors is reached. To evaluate the performance of a model without the j-th variable, the one of the *j* component of *x* is exchanged for a zero. With this, the metrics are computed with the already defined function Metrics and finally by averaging their values.

**Algorithm A2** Reduction algorithm to feature selection	
**Require:**$x_{0}:$ Vector of zeros and ones that indicate whether the regressor is selected, $n_{\min}:$ Minimum number of regressors selected, D: Data (X,y) compatible with *x*, I: Stratified indexes of *D*, with length p=|I|, of the form $\left[I_{1},\ldots ,I_{p}\right]$	
**Ensure:***x*	
1: $\left(x,n\right)\leftarrow (x_{0},|\left\{x=1\right\}|)$	▹ Initialization
2: **while** $n_{\min}<n$ **do**	▹ Recursive stage
3: (w,z)←(∅,SeedsGenerator(I))	
4: **for** jin{x=1} **do**	
5: $x^{*}\leftarrow x$	
6: $x^{*}\left(j\right)\leftarrow 0$	
7: $m\leftarrow \mathrm{Metrics}\left(\left\{x^{*}\right\},z,D,I\right)$	
8: $w_{j}\leftarrow \mathrm{Average}\left(m_{x^{*}}\right)$	
9: Append $w_{j}$ to *w*	
10: **end for**	
11: Let $j^{*}$ be the argument of the maximum value in *w*	
12: $x(j^{*})\leftarrow 0$	
13: n←n−1	
14: **end while**	
